# Metal surface defect detection based on improved YOLOv5

**DOI:** 10.1038/s41598-023-47716-2

**Published:** 2023-11-27

**Authors:** Chuande Zhou, Zhenyu Lu, Zhongliang Lv, Minghui Meng, Yonghu Tan, Kewen Xia, Kang Liu, Hailun Zuo

**Affiliations:** School of Mechanical and Power Engineering, Chongqing University of Science and Technology, Chongqing, 401331 China

**Keywords:** Electrical and electronic engineering, Computer science

## Abstract

During the production of metal material, various complex defects may come into being on the surface, together with large amount of background texture information, causing false or missing detection in the process of small defect detection. To resolve those problems, this paper introduces a new model which combines the advantages of CSPlayer module and Global Attention Enhancement Mechanism based on the YOLOv5s model. First of all, we replace C3 module with CSPlayer module to augment the neural network model, so as to improve its flexibility and adaptability. Then, we introduce the Global Attention Mechanism (GAM) and build the generalized additive model. In the meanwhile, the attention weights of all dimensions are weighted and averaged as output to promote the detection speed and accuracy. The results of the experiment in which the GC10-DET augmented dataset is involved, show that the improved algorithm model performs better than YOLOv5s in precision, mAP@0.5 and mAP@0.5: 0.95 by 5.3%, 1.4% and 1.7% respectively, and it also has a higher reasoning speed.

## Introduction

As an indispensable contributor to national development, metal material enjoys various types and vast application scenarios. However, due to factors such as machine and environment involving in the production process, the wide variety of surface defects are unavoidable, such as crack, inclusion and blister and the like. If those defects are not recognized in time, it would bring potential risks to product quality and safety. Therefore, it is of great significance to improve metal surface defect detection to avoid economic losses and staff casualties. Effective detection of metal surface defects is the key step to ensure the production process safety and product quality. Thus, people have been looking for more efficient and reliable methods to detect metal surface defects in practical production^1^. Traditional methods include visual inspection and manual operation. However, these methods have problems of low accuracy and efficiency, and can be easily affected by subjective factors. With the advancement of science and technology, various new metal surface detection techniques have appeared in recent years. For instance, digital image processing techniques^2^ and deep learning algorithms^3^ are applied to enable automate metal surface defect detection, thus significantly improving detection accuracy and efficiency. Moreover, nondestructive detection techniques including ultrasound^4^, x-ray^5^ and infrared ray^6^ are also contributable to the detection of inner metal defects, effectively guaranteeing the quality and safety of products. In the research of metal surface defects detection based on deep-learning technology, Zhao et al.^7^ introduced RDD-YOLO model and designed a double feature pyramid network (DFPN) to enhance the neck and generate enriched representation so as to deepen the whole network and make a special use of low-level feature. This model has achieved obvious effect in terms of steel surface defect detection. Xie et al.^8^ proposed a surface defect detection algorithm based on feature enhanced YOLO which is suitable for practical industrial applications. An improved feature pyramid network is designed to enhance the spatial location correlation for multi-scale detection layer to achieve high precision. Cheng et al.^9^ introduced a new deep neural network (DNN) with better recognition performance to detect steel surface defect, namely RetinaNet with difference channel attention and adaptively spatial feature fusion (DEA-RetinaNet). Liu et al.^10^ put forward a new Haar-Weibull variance model to detect steel surface defect in an unsupervised manner. This method can detect any type of defect on the even texture and realize an average detection rate of 96.2% in terms of dataset. Yu et al.^11^ introduced a new deep-learning detection network, channel attention and bidirectional feature fusion on a fully convolutional one-stage (CABF-FCOS) network to realize a quick and efficient defect detection of steel strip. However, there are still many problems regarding methods of small target defect detection on the metal surface. For instance, the YOLOv5 algorithm was designed with as many data enhancement techniques taken into account as possible. But, in the practical applications, the robustness of the model is still insufficient when it comes to complicated scenarios or images with high noise, resulting in false or missing detection. YOLOv5 adopts Cross Stage Partial Network as the backbone network, which can extract enriched feature information. However, when dealing with small targets, the network cannot make full use of the low-level feature information because of the giant down-sampling step of CSPNet, thus causing insufficient feature extraction. When several small targets overlap at the same area, YOLO algorithm may not able to detect them correctly. Despite the fact that some measures for improvement have been introduced to deal with the problems mentioned above, including special convolutional structure (like SPPNet^12^, SAM^13^, SSD^14^, SRM-FPN^15^, PAN^16^, ASFF^17^, NAS-FPN^18^), improved loss function and data augmentation, the problems regarding detection accuracy and speed still remain.

To further improve these measures, we introduce an upgraded defect detection model based on YOLOv5s model. In the model training, data augmentation techniques including random rotation, brightness adjustment, sensor noise addition and glass blur are applied to conduct metal surface detection, and the results are finally verified in the GC10-DET augmentation dataset.

1. CSplayer module is introduced to promote the detection of small target while maintaining the light-weighting of the model. The C3 module in the YOLOv5s structure is replaced with CSPlayer module so that the model can adaptively adjust the channel weight, deepen model layers and improve model capability. In comparison with C3module, CSPlayer can achieve better training and reasoning speed while ensuring accuracy. By splitting large convolution kernels into small ones, the model has lower video memory consumption, thus becoming more suitable for training and reasoning under the condition of limited memory.
2. GAMAttention module that combines channel attention and spatial attention is introduced to augment feature expression, improve the detection of small target and better capture the complicated feature of image. The attention weight is modeled into a GAMAttention module, in which the attention weight of each dimension is represented by independent nonlinear function. Compared with other attention mechanism, GAMAttention is superior in flexibility and interpretability and can handle different types of sequence data more efficiently.

## Related work

### Small target detection

In the research of visual detection algorithm based on deep-learning, especially the YOLO series algorithms, small target defect detection cannot be accurately handled in terms of accuracy and speed. Small target is featured with low resolution, blurred image, insufficient information and much noise. Some measures have been introduced to resolve those problems. For example, data augmentation strategy fixes up problems of insufficient information of small target and texture, but increases computing costs. Guo et al.^19^ introduced a new deep-learning model named Small Target CenterNet, which applies the selective small target replication algorithm (SSTRA)and increases the number of small targets through selective over-sampling. Although being conducive to the feature extraction of small targets, it increases additional computation and is easily affected by noise. Wang et al.^20^ proposed a small target detection method (C-SSD) based on improved SSD and realize the feature fusion of compact block through quick connection. The introduction of prediction layer residual and DIoU-NMS further improve detection accuracy. Zhang et al.^21^ introduced a network model structure based on YOLOv5, and added the detection head that is generated from low-level feature layer and high-resolution feature combination image to detect small object. However, this method has higher requirements for training dataset and is not significantly conducive to improving the detection of small targets. Li et al.^22^ put forward a small target prediction head that combines YOLOv5 (GBH-YOLOv5) to detect defects of PV panel. The BottleneckCSP module is introduced to ensure a better accuracy regarding multi-scale targets. The Ghost Convolution is applied to improve reasoning speed of the model and reduce the number of parameters. Those measures mentioned above demonstrate well performance in terms of accuracy and speed, but other problems still exist. For instance, the features of small target cannot be fully extracted, thus causing large errors and insufficient detection of small target. Furthermore, performance gain empowered by those measures are limited by computation costs. Therefore, this paper is intended to find ways to achieve highly accurate metal surface defect detection by improving feature expression and reducing computation.

### Attention mechanism model

In recent years, many researchers have introduced Attention Mechanism into Convolutional Neural Networks to improve target detection capability, and various Attention Mechanisms have gained progress as to the research of small target detection. Due to the complicated background of small target, the Spatial Attention is introduced in the target detection. Through weighing the features of targeted area, the feature extraction network can selectively focus on the targeted area that contains important information and suppress unrelated information, thus decreasing the impact of the background on the detection results and promoting the detection performance of the model. Channel Attention enables the network to focus more on the crucial feature channel and ignore unimportant ones, thus improving the accuracy and robustness of the model and reducing computation and overfitting. Huang et al.^23^ introduced an improved YOLOv3 algorithm Spatial Attention that is based on Gated Channel Transformation and adaptive up-sampling module, and mainly focus on the feature reaction of different location of the image, thereby improving the model’s detection performance of targets. The SEAT-YOLO proposed by Kumar et al.^24^ can detect small shadow area with high accuracy. By integrating Channel Attention and Spatial Attention, the Channel and Spatial Mixed Attention can better focus on the target with relatively less pixels and promote the accuracy of small target detection. Deng et al.^25^ introduced a method to detect small targets in the Optical Remote Sensing, which combines the hybrid domain attention units (HDAUs) of channel attention and spatial attention, thereby improving feature extraction ability and suppress background noise. Self-Attention principally focuses on the dependence relations in the input sequence to pick up the modelling capacity sequence data. Xue et al.^26^ integrated self-Attention and ACmix into YOLOv5. They reduced resource consumption of model and increased the detection accuracy of small targets by combining Global Context Network. Multi-Head Attention primarily coordinates various types of attention mechanisms in order to boost the expression capacity and robustness of the model. Liu et al.^27^ introduced an improved YOLO model based on cross-attention strategy transformer, ensuring better robustness for models with noise input. To sum up, small target detection has been a challenging problem in the YOLO series algorithm. Different attention mechanism models can enhance the detection accuracy of small targets. However, problems still exist, including how to make the model better understand the significance of various areas in the image and the interrelations of different areas, so as to improve the detection accuracy of small targets and how to better improve the model’s performance of real-time and extensibility.

## Methods

### Review of YOLOv5

YOLOv5s is CSPDarknet53, which is a convolutional neural network extracted from the DarkNet framework. CSPDarknet53^28^ reduces the number of parameters and improves model performance by using the cross stage part (CSP) module. Neck: Based on its backbone Network, YOLOv5s applies the Feature Pyramid Network (FPN) technology to construct the feature pyramid. The feature pyramid is made up of multiple feature maps of different scales, which can capture objects of different sizes and provide more information for detectors. Head: The detection head of YOLOv5s consists of three convolution layers and an output layer. The output layer uses sigmoid activation function to process the detection results and outputs information such as detection box, class confidence and classification probability.

### Improved network architecture

**Figure 1:**
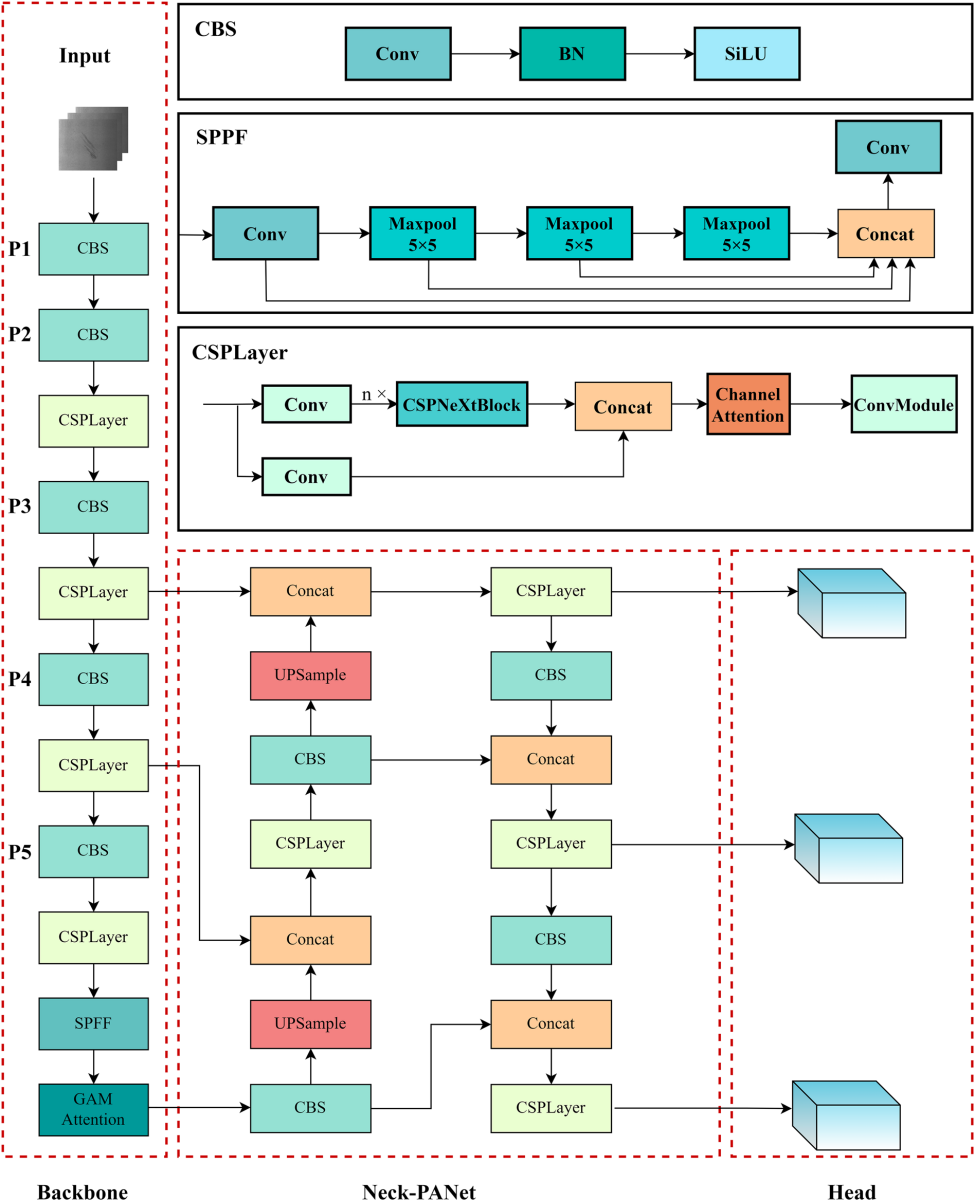
Structure of improved network architecture.

The model structure proposed in this paper is shown in Fig. 1. The main improvements over YOLOv5s are as follows:

1. YOLOv5s takes CSPDarknet as a multi-scale feature extraction network, and includes convolution blocks of 2 steps with its kernel of 6 × 6, three groups of Conv + C3 structures and spatial pyramid pool-fast module. To further improve model performance, we replace the C3 layer with CSPlayer^29^. While maintaining the original model structure, some new characteristics and technologies are utilized to increase the speed and reduce the model size with its precision guaranteed. It will facilitate the detection of small defects on metal surface.
2. The introduction of GAMAttention^30^ enables the model to focus more on the object area, reduce the computation and improve the detection speed and accuracy. The possibility of background interference in metal surface defect image acquisition is relatively high. These functions of GAMAttention can adaptively learn how much each dimension contributes to the output, and are well interpretable.

### CSPlayer module

**Figure 2:**
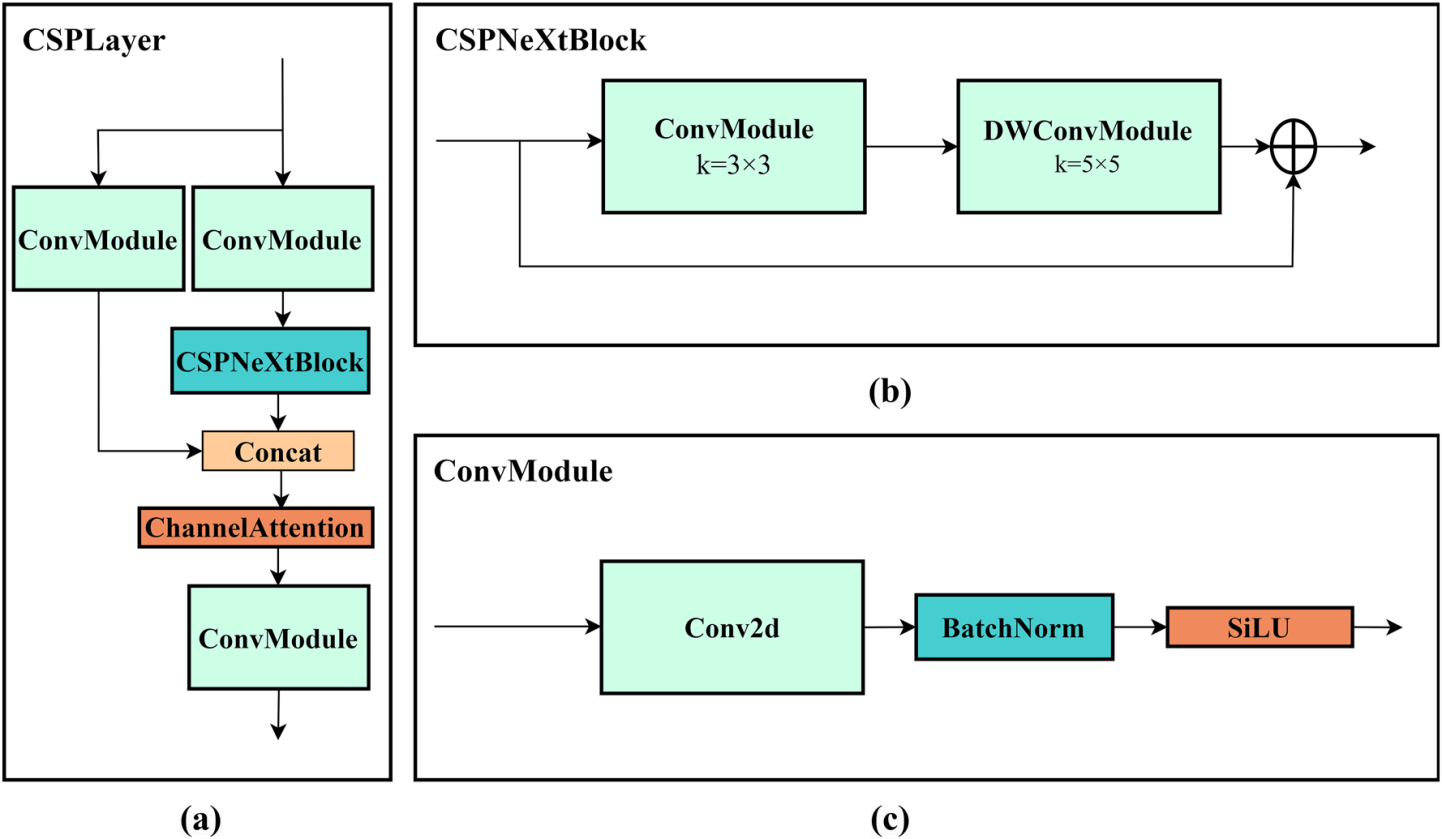
CSPlayer structure: (**a**) CSPlayer module, (**b**) CSPNeXtBlock, (**c**) ConvModule.

Both highly accurate detection results and real-time performance and computing resources need to be taken into account, so as to minimize redundant expression and reduce the computation. This paper introduces CSPlayer module and gives full play to its advantages to achieve the goal of better feature expression ability and less computation. The structure is shown in Fig. 2 The module consists of three Convmodules, n CSPNext blocks and one Channel Attention module. The ConvModule consists of a layer of 3 × 3 Conv2d, BatchNorm, and SiLU activation functions.

**Figure 3:**
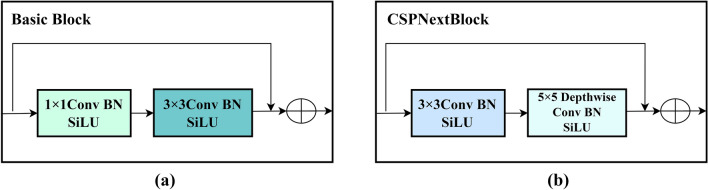
Convolution structure: (**a**) Basic block. (**b**) CSPNextBlock.

In YOLOv5s, CSPDarknet53 adopts a multi-branch structure similar to ResNeXt, where each branch separately processes a portion of the input and then splices the parts together, thus reducing computation and memory occupation and improving the ability of feature representation. In the YOLOv5s structure, the C3 module can help the model to better capture multi-level feature information of the object. It can extract both details and context information of the object at the same time by employing multiple different convolution kernel sizes and channel numbers. The idea of CSP is followed in CSPlayer. Darknet (Fig. 3 (a)) uses a Basic Block of 1 × 1 and 3 × 3 convolution and adds the Depthwise convolution of large kernel (Fig. 3 (b)). The structure here uses 5 × 5 DW convolution to achieve fewer parameters and bring about more receptive field. CSPlayer connects the output features of all previous layers as the input of the next layer to maximize cardinality. Based on the idea of gradient information combination, let high cardinality and sparse connectivity enhance the learning ability of the network. Replacing the Pointwise Convolution with a Depthwise Separable Convolution can be decomposed into two smaller operations. Firstly, Depthwise Convolution is employed to convolution different input channels respectively, and then Pointwise Convolution is used to combine the above outputs. In this way, the overall effect is similar to that of a Pointwise Convolution, but the computation and model parameters are greatly reduced.

### Enhanced backbone with GAMAttention

**Figure 4:**
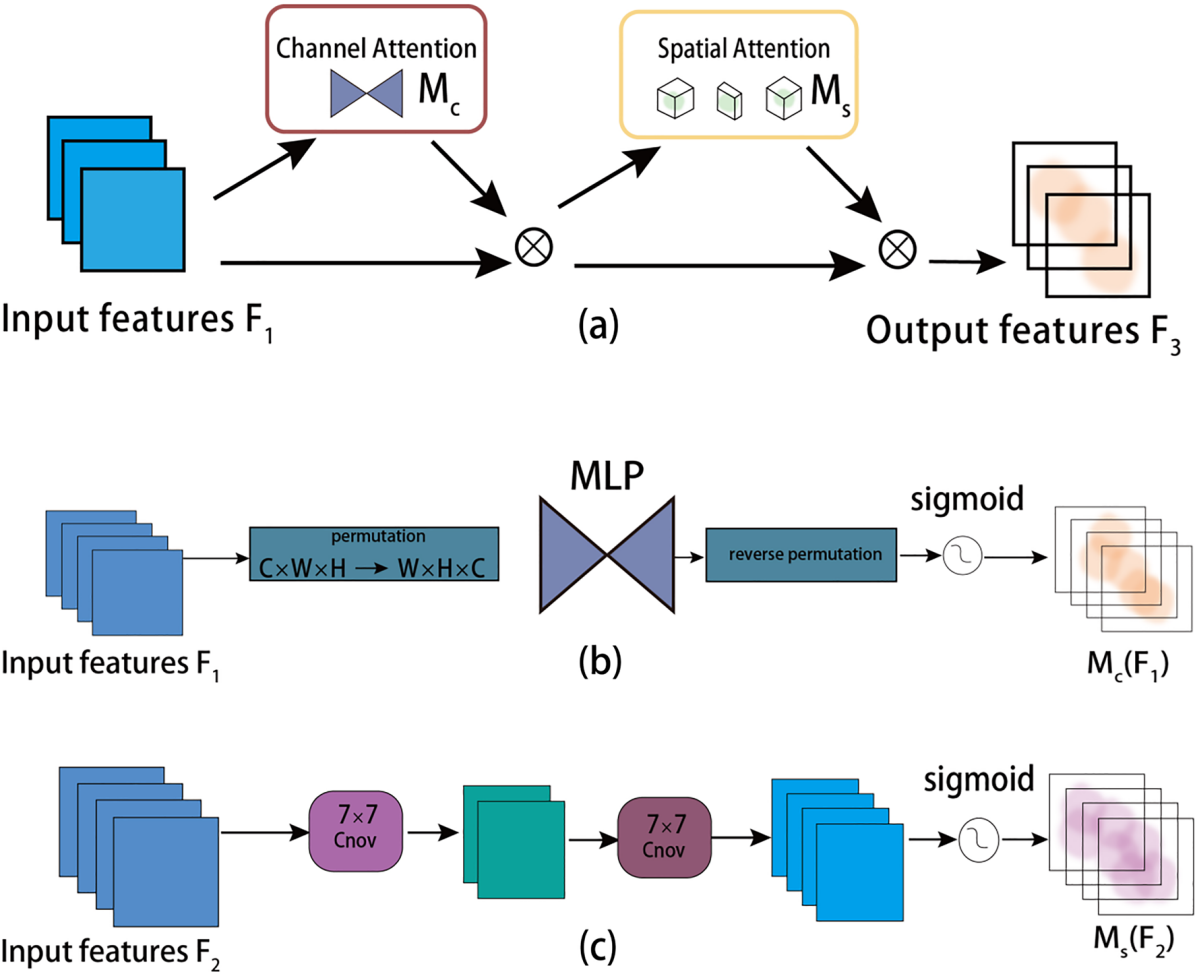
(**a**) The overview of GAM. (**b**) Channel attention submodule. (**c**) Spatial attention submodule.

Small targets usually have a small number of pixels, which makes it difficult for detectors to recognize. The GAMAttention introduced in this paper can make the model focus more on small target location rather than background information and improve its detection of small targets. It can also adaptively learn the size and location information of each small target to further improve the accuracy of small target detection. GAMAttention is a global attention mechanism, and its overall structure (Fig. 4 (a)) uses the channel attention mechanism and the spatial attention mechanism. For the input feature map, GAMAttention first performs dimension conversion, adjusting the dimensions of the feature map. The purpose of doing this is to reduce computational complexity and the number of parameters. The feature map after dimension conversion is input into a multi-layer perceptron (MLP), which further processes the feature map (Fig. 4 (b)). Then, the processed feature map is transformed back to its original dimension. Finally, it is output after being processed by the Sigmoid function. Compared with Spatial Attention Module (SAM), GAMAttention mainly uses convolutional operations to handle channel attention (Fig. 4 (c)). First, reduce the number of channels and computation by applying a convolution operation with a kernel size of 7. Then, increase the number of channels by applying another convolution operation with a kernel size of 7 to maintain consistency in the number of channels. Finally, apply the Sigmoid function to output the weights of channel attention. Formulas 1 and 2 indicate that given input feature mapping F1 ∈ RC × H × W, intermediate state F2 and output F3 are defined as:

1 $$ \begin{array}{*{20}c} {{\text{F}}_{2} = {\text{M}}_{{\text{C}}} \left( {{\text{F}}_{1} } \right) \otimes {\text{F}}_{1} } \\ \end{array} $$

2 $$ \begin{array}{*{20}c} {{\text{F}}_{3} = {\text{M}}_{{\text{s}}} \left( {{\text{F}}_{2} } \right) \otimes {\text{F}}_{2} } \\ \end{array} $$

Mc and Ms are channel attention graph and space attention graph respectively.$\otimes$ Represents the multiplication of element-wise. The overall process is illustrated in Fig. 4 It can transfer information between multi-layer convolutional feature graphs and enhance the expression of targets by learning feature representations at different levels. After the input data is projected through multiple fully connected layers, the attention weight of each dimension is calculated by using a nonlinear function. Finally, the weighted average of the attention weight of all dimensions is taken as the output. This mechanism helps the model to ignore areas that are not important, thus improving the robustness of the model to changes in scene and lighting. Through the GAMAttention, the model can adaptively learn the scale and location information of each target, and maintain a good detection effect even in the case of scale change or translation transformation. During the training, the GAMAttention can suppress features unrelated to the target and focus more on features relevant to the target, thus reducing noise interference and overfitting risk. In this way, the model can better understand the target information and improve the detection accuracy of small targets.

## Experiment

### Dataset

**Figure 5:**
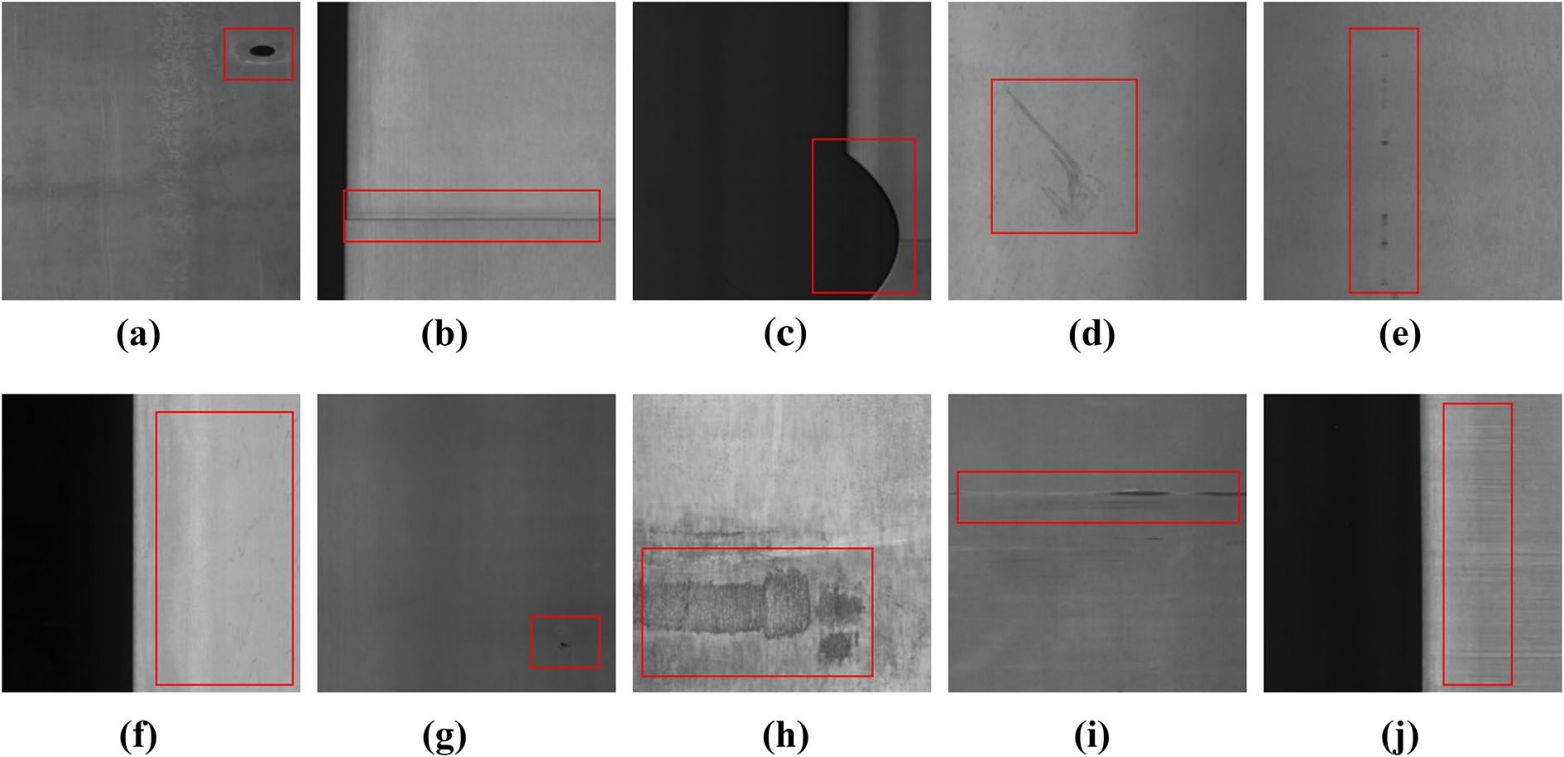
Ten types of surface defects in GC10-DET dataset: (**a**) punching hole, (**b**) welding line, (**c**) crescent gap, (**d**) water spot, (**e**) oil spot, (**f**) silk spot, (**g**) inclusion, (**h**) rolled pit, (**i**) crease, (**j**) waist folding.

The dataset adopted in the experiments is the GC10-DET dataset^31^, which contains ten types of metal defects, namely, Punching hole (Ph), Welding line (Wl), Crescent gap (Cg), Water spot (Ws), Oil spot (Os), Silk spot (Ss), Inclusion (In), Rolled pit (Rp), Crease (Cr), Waist folding (Wf). In order to restore the possible complexity of metal defect detection, we augmented the dataset by random rotation, brightness adjustment, adding sensor noise and glass blurring, etc. The augmented images are shown in Fig. 5 The total number of images in the expanded dataset is 4508. And the dataset is divided into train set, validation set and test set in the ratio of 8:1:1. The training set has 3608 images and the test set and validation set have 450 images each.

### Experimental environment and training parameters

The configuration of the host computer used in this experiment includes a graphics card of RTX 3060, CPU of Intel(R) Core (TM) i5-12400F, and the Windows 10 operating system. PyTorch 1.12.1, Python 3.8 and CUDA12 are employed in the experiment. The training parameters used in the experiment are shown in Table 1.

**Table 1:** Training parameters.

Parameter	Value
Learning rate	0.01
Batch size	8
Image size	640*640
Weight decay	0.0005
Momentum	0.937
Epoch	300

### Evaluation metric

A variety of metric are used in this research to evaluate the performance of the model, which include precision (P), recall (R), mean average precision (mAP), frames per second (FPS) , and model size. These metrics provide a comprehensive picture of the model's performance in the classification task.

Precision is the proportion of the number of samples correctly predicted as true positives to the number of all samples predicted as true positives by the model, and the formula for computation is as follows:

3 $$ \begin{array}{*{20}c} {Precision = \frac{{{\text{TP}}}}{{{\text{TP}} + {\text{FP}}}}} \\ \end{array} $$

Recall is the share of the number of samples that the model correctly predicts as true positives to the number of all samples that are actually true positives, and it can be calculated by the formula below:

4 $$ \begin{array}{*{20}c} {Recall = \frac{{{\text{TP}}}}{{{\text{TP}} + {\text{FN}}}}} \\ \end{array} $$

F1-Score is the metric that comprehensively combines Precision and Recall and the formula for computation is given below:

5 $$ \begin{array}{*{20}c}    {F1 = \left( {\frac{2}{{{\text{Recall }}^{{ - 1}}  + {\text{Precision }}^{{ - 1}} }}} \right) = 2 \cdot \frac{{{\text{Precision}} \times {\text{Recall}}}}{{{\text{Precision}} + {\text{Recall }}}}}  \\   \end{array}  $$

mAP and AP are metrics used for the evaluation of multi-category classification problems. mAP is the average of AP values for all categories, while AP is calculated separately for each category. They are calculated as shown below:

6 $$ \begin{array}{*{20}c} {AP = \mathop \smallint \limits_{0}^{1} {\text{P}}\left( {\text{R}} \right){\text{dR}}} \\ \end{array} $$

7 $$ \begin{array}{*{20}c} {mAP = \frac{{\mathop \sum \nolimits_{{{\text{j = }}1}}^{{\text{s}}} {\text{AP}}\left( {\text{j}} \right)}}{{\text{S}}}} \\ \end{array} $$

In the formula, S denotes the total number of categories. FPS refers to the number of frames that the model can process per second, and model size indicates the amount of storage space occupied by the model. All these metrics are of great significance for the evaluation of the performance and adaptability of the model.

## Experimental results

In order to verify the effectiveness of the various modules introduced in this paper, the ablation experiment is designed as shown in Table 2. In Scheme I, the model is the original YOLOv5s model; in Scheme II, the C3 module is replaced with CSPLayer module in the network structure; in Scheme III, GAMAttention is introduced; in Scheme IV, CSPLayer + GAMAttention is introduced on the basis of the original YOLOv5s. The following are the arguments for each improvement based on the experimental results:

1. Influence of CSPlayer

The results of Scheme II in Table 2 show that the recall, mAP@0.5 and mAP@0.5:0.95 improve by 0.7%, 0.1% and 1.3%, respectively. Accordingly, all the small target defects are detected by the model with a higher confidence. This indicates that the detailed information and localization information of small targets can be effectively extracted by the CSPlayer module, reducing missing detection of target.

(2) Influence of GAMAttention

The results of Scheme III in Table 2 demonstrate that all the metrics gained increase to some extent, except for a slight drop in recall. However, the decrease in recall may be attributed to the introduction of local interaction mechanisms by the GAMAttention module, which may occasionally overlook small targets, resulting in a slight decrease in recall. This trade-off needs to be fine-tuned and optimized for specific tasks to meet performance requirements. In the meanwhile, in comparison to the original model, the model that added GAMAttention module achieved progress in terms of the detection accuracy of small targets. The performance improvement observed may be attributed to GAMAttention, which helps reduce the model's sensitivity to local errors in the image. This means that even if the targets are small, the model can locate them more accurately, thereby improving the accuracy of object detection.

(3) Influence of CSPlayer and GAMAttention

The results of Scheme IV in Table 2 show that the precision, mAP@0.5 and mAP@0.5: 0.95 improve by 5.3%, 1.4% and 1.7%, respectively. Therefore, using CSPlayer and GAMAttention to improve the YOLOv5s model can enhance the performance of small object detection, with high accuracy and computational efficiency, which is of great significance for practical applications of small object detection tasks.

**Table 2:** Ablation experiments.

Model	Precision(%)	Recall(%)	mAP@0.5(%)	mAP@0.5:0.95(%)
YOLOv5s	88.5	77.1	81.4	53.8
YOLOv5s + CSPLayer	88.1	77.8	81.5	55.1
YOLOv5s + GAMAttention	91.7	77.0	82.4	53.9
YOLOv5s + CSPLayer + GAMAttention	93.8	76.0	82.8	55.5

## Results comparison of different attention mechanisms

**Figure 6:**
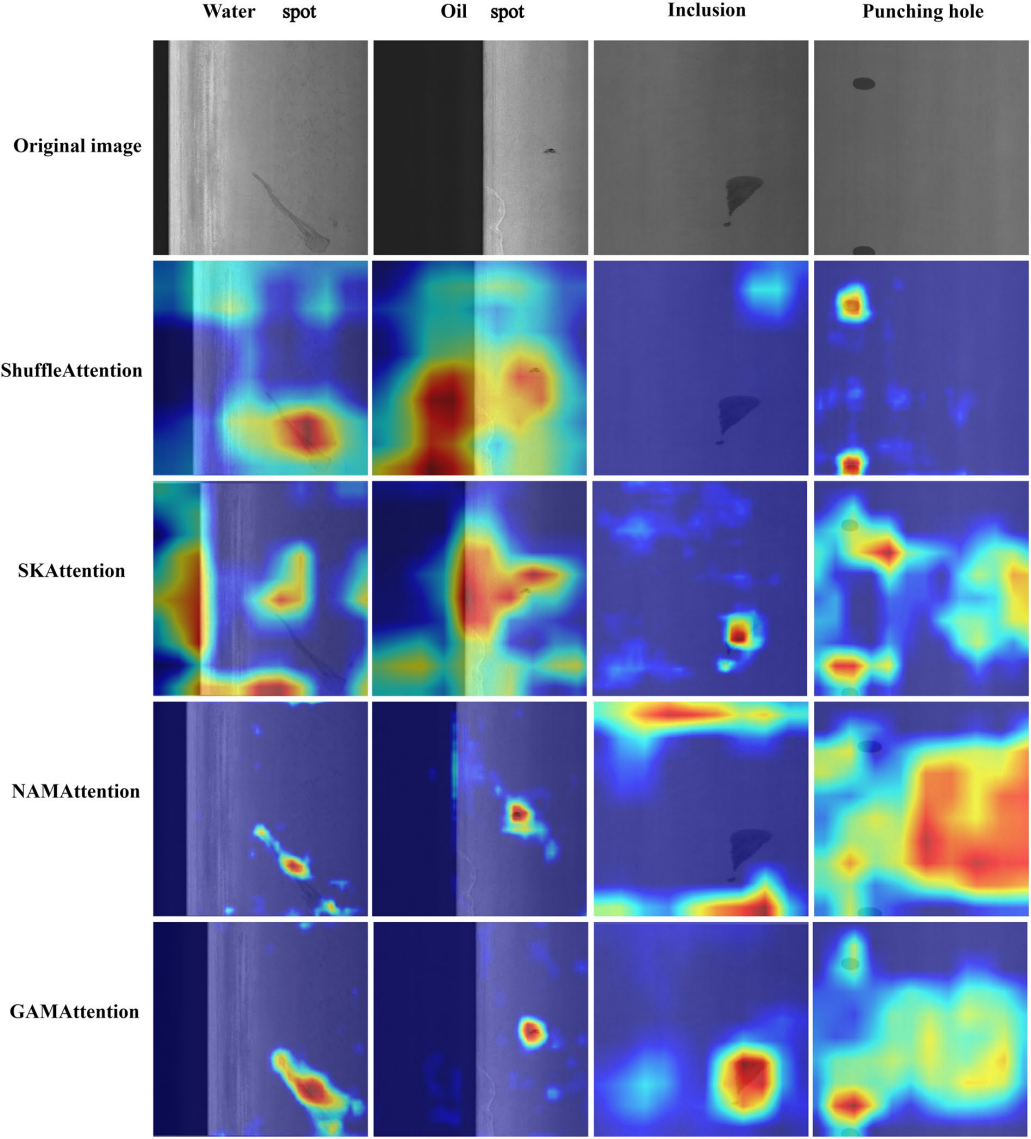
Heatmaps of attention mechanism comparison.

Comparative experiments were carried out by introducing different attention mechanisms. According to the experimental results (Table 3), it was found that among these attention mechanisms, ShuffleAttention performs better when dealing with large-scale targets, such as Welding line. In the comparative experiment, the mAP@0.5 reached 96.5%, achieving the highest detection accuracy compared to other attention mechanisms. From Fig. 6, it can be seen that ShuffleAttention has better performance in Water spot detection but missed Inclusion. ShuffleAttention may be more sensitive to target scale. For small object detection, the characteristics of small objects may make it difficult for ShuffleAttention to capture enough relevant information, thereby reducing performance. However, for Welding line, these targets may have larger scales, making it easier to capture relevant information. The SKAttention module is an attention mechanism that combines group convolution, spatial attention, and channel attention. Its design goal is to improve object detection performance while reducing computational complexity. However, although the SKAttention module performs well in the Water spot detection task, achieving an mAP@0.5 of 84.5%, it performs poorly in detecting small objects at different positions (Fig. 6). This is because different objects usually have low contrast, and the model needs more spatial context information to accurately detect them. NAMAttention uses the contribution factor of weight to enhance the effectiveness of attention. It performs well in Crescent gap detection, but generally performs poorly in Rolled pit and Crease detection. NAMAttention may perform better in tasks with less noise or simpler tasks, while performing worse in complex or high-noise environments. It may also be influenced by the scale and shape of the target. Crescent gap may have certain shape characteristics that make it easier for NAMAttention to capture relevant information. For Rolled pit and Crease, the shape and characteristics of the target may not match well, resulting in poor performance. According to the results of attention mechanism comparison experiment (Table 3) and the heatmap (Fig. 6), it can be seen that Water spot, Oil spot, Inclusion, and Punching hole have higher defect detection accuracy, indicating that GAMAttention has higher attention to small targets, which can better capture local information in the image and improve the detection rate of small targets. GAMAttention reduces the number of channels through convolution, reducing computational complexity and improving detection speed. Therefore, based on the experimental results, it can be concluded that introducing GAMAttention attention mechanism is suitable for small target detection, which can improve the accuracy of the model and has faster detection speed compared to other attention mechanisms.

**Table 3:** Attention mechanism comparative experiment.

Types	AP(%)										mAP@0.5(%)	mAP@0.5:0.95(%)	FPS (f/s)
	Ph	Wl	Cg	Ws	Os	Ss	In	Rp	Cr	Wf			
ShuffleAttention	98.1	96.5	90.5	77.0	81.2	80.4	48.9	93.3	70.2	85.7	82.2	53.4	72.4
SKAttention	95.0	93.1	98.3	84.5	88.0	86.7	61.0	53.4	55.4	83.2	79.9	49.8	76.3
NAMAttention	94.4	89.6	98.8	81.1	87.8	81.9	49.4	51.0	50.0	77.8	76.2	43.8	78.0
OURS	95.7	95.8	98.9	83.2	90.1	90.1	66.3	58.9	62.4	87.0	82.8	55.5	79.4

### Comparison experiment

To validate the performance of the network model proposed in this paper for the small target detection, we conducted extensive experiments and compared other existing detection models. Faster R-CNN, YOLOv5s, YOLOv5l, YOLOv5m, and YOLOv5x were separately used for detection on the augmented GC10-DET dataset.

According to the results of the comparison experiment (Table 4), Faster R-CNN presents poor performance on the GC10-DET augmented dataset and is lower in confidence compared to the detection results of other algorithms, indicating that Faster R-CNN does not work well in terms of small targets detection. Compared with several algorithm models, our proposed model effectively improves the detection accuracy for various defects, in which the detection accuracy AP value for Silk spot that contains a large number of small targets reaches 90.1%, and the detection accuracy AP values for Punching hole, Welding line, and Crescent gap reach 95.7%, 95.8% and 98.9% respectively, which is superior to other algorithm models.

**Table 4:** Algorithm model comparative experiment.

Model	AP(%)										mAP@0.5(%)	mAP@0.5:0.95 (%)	FPS (f/s)
	Pu	Wl	Cg	Ws	Os	Ss	In	Rp	Cr	Wf			
Faster R-CNN	90.4	44.9	97.5	81.2	81.7	81.2	62.8	60.7	54.5	90.3	74.5	32.5	23.0
YOLOv5s	92.9	96.1	97.8	81.6	88.9	89.4	62.9	55.5	62.7	86.1	81.4	53.8	78.1
YOLOv5m	95.4	95.4	98.5	82.2	87.6	89.3	65.6	53.1	65.1	86.8	81.9	54.8	76.9
YOLOv5l	94.3	94.5	97.9	84.3	89.8	89.7	65.5	61.2	66.6	83.2	82.7	54.3	76.3
YOLOv5x	94.1	93.9	98.1	83.5	90.5	87.4	65.1	52.7	63.3	85.0	81.4	54.5	75.3
OURS	95.7	95.8	98.9	83.2	90.1	90.1	66.3	58.9	62.4	87.0	82.8	55.5	79.4

**Figure 7:**
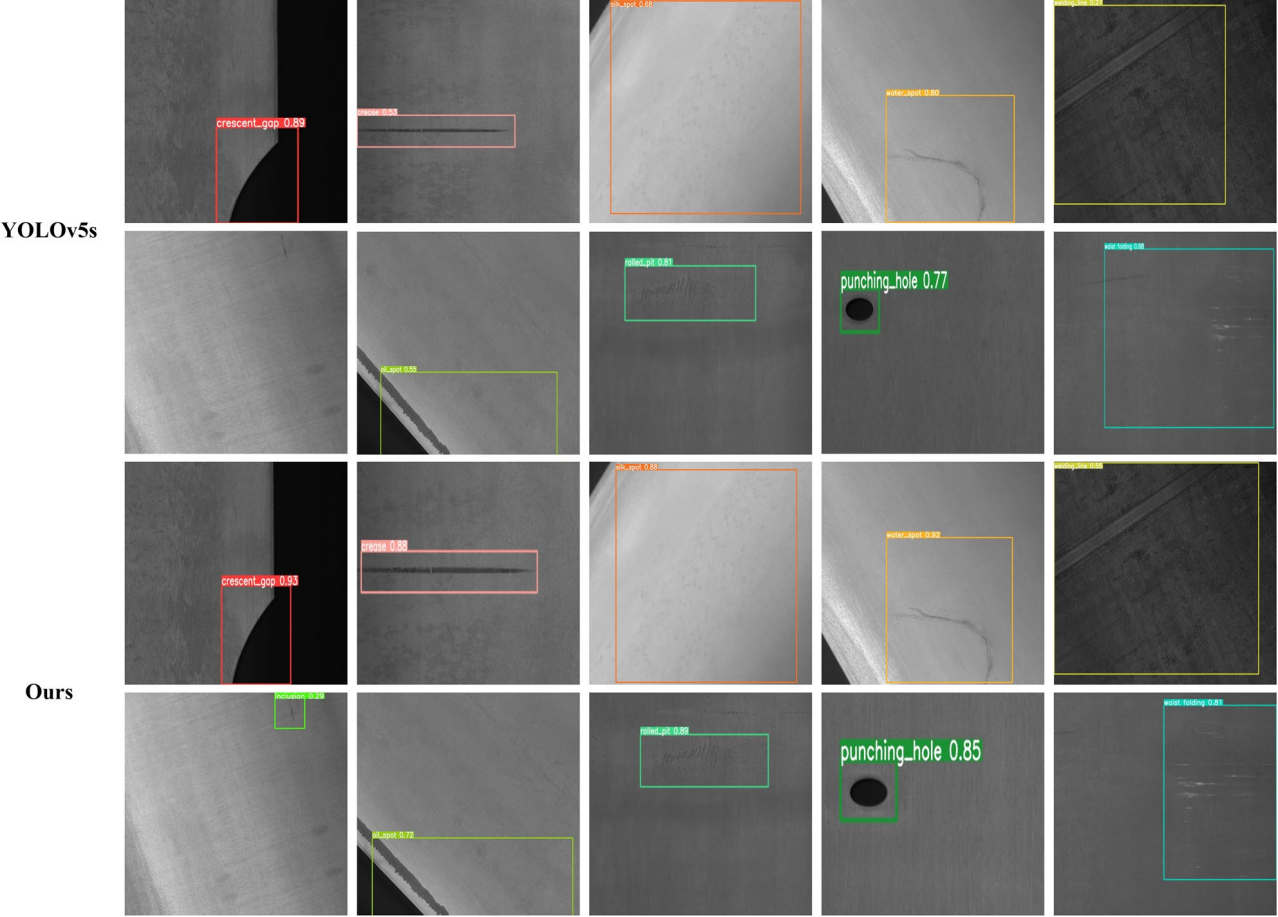
Detection results.

In the performance of the improved model, the mAP@0.5 and mAP@0.5:0.95 of the proposed model reached 82.8% and 55.5%, respectively, 1.4% and 1.7% higher compared to the original YOLOv5s. In terms of detection speed, this model achieves 79.4 FPS, which shows that the improvements made in this paper for the feature of small targets are effective. In addition, other algorithms are confronted with missing detection, and only the model proposed in this paper correctly detected all the targeted defects. From the detection results (Fig. 7), it can be found that the improved model has more accurate localization of target defects, which improves the accuracy of the model. In a word, the network model we propose shows high accuracy and reliability when it comes to small target detection. It promotes the detection speed of metal surface small targets and better meets the industrial needs of high-speed and automate metal surface detection, offering support and assistance for practical applications.

## Conclusion

The detection model introduced in this paper is specifically intended to improve the detection accuracy and speed of small target defects in the GC10-DET augmented dataset. The model improves the YOLOv5s network structure, which mainly includes the following aspects: first, the C3 module in YOLOv5s is replaced with the CSPlayer module, which enables the model to adaptively adjust the channel weights as needed, thus improving its flexibility and adaptability; second, the GAMAttention is introduced to help reduce the computation while improving detection accuracy and speed. The experimental results show that, in comparison with other detection model, this model performs better on the GC10-DET augmented dataset, and can effectively reduce false and missing detection. In addition, it can also meet the demand for lightweight deployment, which is suitable for industrial scenarios such as metal material surface defect detection. However, we also found that there is still room for improvement in detection accuracy for defects like Rolled pit of relatively large size, and further optimization of the algorithm design is needed to resolve this problem.

## Data Availability

The datasets used and analyzed during the current study are available from the corresponding author upon request.
